# Dietary Supplementation of Myo-Inositol, Cocoa Polyphenols, and Soy Isoflavones Improves Vasomotor Symptoms and Metabolic Profile in Menopausal Women with Metabolic Syndrome: A Retrospective Clinical Study

**DOI:** 10.3390/medicina60040598

**Published:** 2024-04-04

**Authors:** Giampaolo Mainini, Salvatore Ercolano, Raffaella De Simone, Irene Iavarone, Rosalia Lizza, Mario Passaro

**Affiliations:** Società Campano Calabro Apulo Lucana di Ginecologia ed Ostetricia (S.C.C.A.L.), 80133 Naples, Italy

**Keywords:** menopause, metabolic syndrome, vasomotor symptoms, myo-Inositol, cocoa polyphenols, soy isoflavones

## Abstract

*Background and Objectives:* Hormonal changes physiologically occurring in menopausal women may increase the risk of developing metabolic and vasomotor disturbances, which contribute to increase the risk of developing other concomitant pathologies, such as metabolic syndrome (MetS). *Materials and Methods:* Retrospective data from 200 menopausal women with MetS and vasomotor symptoms taking one sachet per day of the dietary supplement INOFOLIC^®^ NRT (Farmares srl, Rome, Italy) were collected. Each sachet consisted of myo-Inositol (2000 mg), cocoa polyphenols (30 mg), and soy isoflavones (80 mg, of which 50 mg is genistin). Patients recorded their symptoms through a medical questionnaire at the beginning of the administration (T0) and after 6 months (T1). *Results:* We observed an improvement in both the frequency and the severity of hot flushes: increased percentage of 2–3 hot flushes (28 at T0 vs. 65% at T1, *p* value < 0.001) and decreased percentage of 4–9 hot flushes (54% at T0 vs. 18% at T1, *p* value < 0.001). Moreover, symptoms of depression improved after supplementation (87% at T0 vs. 56% at T1 of patients reported moderate depression symptoms, *p* value < 0.001). Regarding metabolic profile, women improved body mass index and waist circumference with a reduction in the percentage of overweight and obesity women (88% at T0 vs. 51% at T1, *p* value = 0.01; 14% at T0 vs. 9% at T1, *p* value = 0.04). In addition, the number of women suffering from non-insulin dependent diabetes reduced (26% at T0 vs. 16% at T1, *p* value = 0.04). *Conclusions:* These data corroborate previously observed beneficial effects of the oral administration of myo-Inositol, cocoa polyphenols, and soy isoflavones against menopausal symptoms in the study population. Considering the promising results of the present study, further prospective controlled clinical trials are needed to deeply understand and support the efficacy of these natural compounds for the management of menopausal symptoms.

## 1. Introduction

Menopause generally occurs as a normal phase of biological aging in women between 45 and 55 years old. It is defined after 12 consecutive months without menstruation due to physiological modifications of hormonal levels [1,2]. In some circumstances, physio-pathological causes or clinical interventions may be responsible for induced menopause, exposing women to symptoms overlapping with those of physiological menopause. 

Despite diagnosis of menopause being clinical, physiological menopause arises from the ovarian follicular depletion that corresponds to low levels of estrogens and high serum concentration of follicle-stimulating hormone (FSH) [3]. The occurrence of menopause may be preceded by a period of menopausal transition by several years that can be primarily identified by (i) increasing irregularity of the menstrual cycle, (ii) hot flushes, (iii) and severe night sweats [4]. During menopause, 85% of women may experiment numerous and various symptoms related to the occurring hormonal modifications that can vary substantially from person to person [4]. Such physiological hormonal alterations can affect physical, emotional, mental, and social well-being contributing to the onset of disturbances in sleep/mood, vasomotor symptoms (VMS), urogenital atrophy, osteopenia and osteoporosis, psychiatric disorders, sexual dysfunction, cardiovascular diseases, metabolic disorders, and obesity [5]. 

Estrogen deficiency and dysregulated lipid metabolism occurring during menopause may expose such women to a higher risk of developing cardiovascular diseases as well [6]. The loss of the protective role of estrogens and the increased circulating androgen levels result in changes to body fat distribution and development of abdominal obesity. Consequently, this increased visceral adipose tissue synthesizes and secretes bioactive substances such as adipocytokines, proinflammatory cytokines, reactive oxygen species, prothrombotic, and vasoconstrictor factors that can affect cardiometabolic health. In addition, during menopause, circulatory Low-Density Lipoprotein Cholesterol (LDL-C) cannot be utilized to synthesize estrogens, resulting in the loss of the protective role of estrogens on the cardiovascular system, thus correlating with higher levels of LDL-C and enhanced cardiovascular risk [7]. Actually, recent works revealed a crucial role of serum levels of High-Density Lipoprotein Cholesterol (HDL-C) as a strong predictor of cardiovascular events. In particular, a recent work by Samar R. El Khoudary et al. [8] highlighted that higher levels of HDL-C are associated with a higher risk of carotid plaque score presence, which is a strong predictor of major adverse cardiovascular events. As concluded by the authors of the study, their results may support the importance of assessing how the menopause transition might impact HDL quality and how that might impact women’s risk of CVD later in life [8].

For this reason, menopause is considered as a risk factor for the development of cardiometabolic diseases, such as metabolic syndrome (MetS), type 2 diabetes, and cardiovascular diseases. MetS, in particular, is defined as the co-existence of interdependent factors including insulin resistance, abdominal obesity, dyslipidemia, and hypertension.

Besides metabolic alterations, VMS are among the primary manifestations of menopause and often considered as the cardinal symptoms. They consist of hot flushes and night sweats predominantly experienced around the head, neck, chest, and upper back. Thurston and colleagues reported in a previous study that 60% to 80% of women already experience VMS during the menopausal transition [9], and these symptoms may last more than 7 years [10] and persist for over a decade [10]. Hot flushes, in particular, can be very disruptive to women’s quality of life [11], impairing sleep, and affecting mood [9,12,13] and daily activities [14]. In addition, mild cognitive impairment and humoral symptoms are characteristic of the menopausal period, typified by symptoms such as anger, irritability, anxiety, depression, sleep disturbance, loss of concentration, and loss of self-esteem/confidence [15]. The occurrence of VMS after menopause may also be associated with an increased risk of developing cardiovascular diseases. In particular, severity rather than frequency of VMS may correlate with increased risk of cardiovascular diseases [16].

Hormonal replacement therapy (HRT) has been considered as one of the first-line treatments for menopausal symptoms [17]; however, its use still remains controversial. HRT is appropriate for managing vasomotor and genitourinary symptoms; indeed, treatments with combined estrogen and progestin regimens reduce the frequency and severity of hot flushes and night sweats by around 75% [18]. Nonetheless, cardiovascular diseases, cognitive impairment, and depression may require further interventions [4]. Due to the risks and the undesirable effects associated with the use of HRT (such as a higher risk of developing breast cancer, weight gain, water retention, vaginal bleeding, mastalgia, headache), many patients prefer to not undergo HRT [19,20,21]; instead, they prefer alternative strategies. Furthermore, HRT is typically prescribed only at the lowest effective dose in short treatment regimens. For all these reasons, about 51% of menopausal women use complementary and alternative medicine, with approximately 60% reporting favorable efficacy in treating menopausal symptoms [19]. In this context, food supplementation and natural therapies, due to their efficacy and excellent safety profile, typically benefit from a higher rate of patient compliance compared to HRT [22]. 

Among natural molecules with applications in menopause [3], the soy extracts or soy isoflavones are effective for counteracting VMS and promoting a balanced metabolism of lipids [23]. Such extracts belong to the class of phytoestrogens that are biologically active plant-derived compounds similar to 17β-estradiol, which have estrogen-like properties [24]. Beneficial properties of phytoestrogens are thought to stem from the higher selectivity of genistein for estrogen receptor (ER)-β vs. ER-α [25]. The reason behind this hypothesis is that estrogens binding to ER-α are considered to be responsible for the majority of side effects associated with estrogen therapy. Among isoflavones, genistin is hydrolyzed into genistein (the bioactive aglycone) by phlorizin hydrolase (a small intestine brush-border lactase) or undergoes further modifications by gut microflora before absorption [26,27]. In particular, the oral bioavailability of genistin results greater than that of genistein [28]. Overall, considering evidence in the literature, isoflavones have been demonstrated to alleviate VMS, reduce spinal bone loss, and decrease hypertension and glycemia [23], thus reducing factors involved in the risk of developing MetS.

Cocoa polyphenols are natural substances present in the cocoa powder obtained from the *Theobroma cacao* tree. These molecules exert a tonic vascular and metabolic effect, perform antioxidant functions, and regulate mood [29]. Various studies have revealed a crucial role of polyphenols for inducing long- and short-term estrogen-dependent changes in menopause. Cocoa polyphenols supplementation may improve blood flow and blood pressure, reducing the risk of occurrence of cardiovascular diseases [30]. Furthermore, cocoa polyphenols’ ability to modulate synthesis and production of nitric oxide, contributes to increase endothelial function, leading to improved arterial blood pressure, reduced levels of cholesterol and blood glucose, especially in older women [31,32].

Myo-inositol (myo-Ins) is a polyol, which acts as a second messenger of various hormones, including insulin [33]. Myo-Ins stimulates glucose cellular uptake from the bloodstream thus regulating glycemic levels and insulin signaling pathway [33]. Previous works revealed that its supplementation, in a dosage of 2 g/day, improves insulin resistance, blood pressure, and lipid profile in a small cohort of postmenopausal women affected by MetS in combination with a low-energy diet versus a control group who only undertook a low-energy diet [34,35]. 

A recent randomized clinical study by D’Anna and colleagues was conducted in post-menopausal women aged between 50 and 60 who were orally administered with cocoa polyphenols, myo-Ins, and soy isoflavones. This study demonstrated the positive effects of such dietary supplementation on blood sugar levels, blood lipid levels, and bone resorption [36], thus displaying the combination of such natural molecules as a promising approach in the management of menopausal symptoms. 

Considering the prior literature, the present study collected data from 200 menopausal women with MetS and assuming a dietary supplement based on cocoa polyphenols, myo-Ins, and soy isoflavones, in order to evaluate the effects of these combined molecules on reducing typical VMS and metabolic menopausal alterations. The effects of the dietary supplement were evaluated by comparing results after 6 months of administration with the baseline; however, the lack of a control group without any treatments may represent a limitation of this study.

## 2. Materials and Methods

### 2.1. Study Design and Patients

The authors collected data from 200 menopausal patients with a diagnosis of MetS based at least on 3 characteristic symptoms: (i) excess abdominal weight (waist circumference more than 35 inches; (ii) hypertriglyceridemia (triglyceride levels ≥ 150 mg/dL); (iii) low levels of HDL-C (HDL-C < 50 mg/dL); (iv) elevated blood sugar levels (fasting blood sugar ≥ 100 mg/dL); (v) high blood pressure (systolic blood pressure values ≥ 130 mmHg and/or diastolic ≥ 85 mmHg or higher). 

Informed consent was obtained from all individual participants. Patients were followed at the Gynecology and Obstetrics clinics of Società Campano Calabro Apulo Lucana (S.C.C.A.L., Naples, Italy), according to the Guidelines for Good Clinical Practice and the Declaration of Helsinki. The retrospective study was approved by the Ethics Committee of Società Campano Calabro Apulo Lucana (protocol number 13/2023/SCCAL) and registered on ClinicalTrials.gov (ClinicalTrials.gov identifier NCT06057896, 28 September 2023).

All retrospective collected data were anonymized prior to access by the authors. All the analyzed data were obtained from menopausal women between 45 and 55 years with a diagnosis of MetS. Another inclusion criterion was having experienced at least 3 months of hot flushes; in addition, the majority of the patient cohort reported changes in mood with reduced attention and memory and a constant feeling of irritation, fatigue, and emotional instability, all symptoms related to typical menopausal hormonal changes. Exclusion criteria included (i) pre-existing cardiovascular morbidities, (ii) pre-existing psychiatric disorders, (iii) undergoing concomitant treatment regimens. In addition, during the entire period of this study, no one patient assumed other medical therapies that could alter or influence the results of this study (i.e., metformin). 

All women assumed for 6 months the dietary supplement containing myo-Ins, cocoa polyphenols, and soy isoflavones (INOFOLIC^®^ NRT, Farmares srl, Rome, Italy). The treatment consisted of one sachet per day (2000 mg of myo-Ins, 30 mg of cocoa polyphenols, 80 mg of soy isoflavones of which 50 mg of genistin), taken separately from meals and dissolved in a glass of water. 

### 2.2. Evaluation of Menopausal Symptoms

At the beginning of the treatment (T0) and after 6 months (T1), patients recorded their symptoms and the eventual variations through the use of a medical questionnaire, which was provided by physician. The patients self-reported VMS in terms of hot flushes and sweats, by evaluating these parameters both in terms of number of episodes (none, 2–3 episodes per 24 h, 4–9 episodes, more than 10 episodes), and severity (none, mild, moderate, severe). 

In addition, the questionnaire also measured the extent of symptoms of depression (none, mild, moderate, severe). A further section of the medical questionnaire recorded metabolic parameters, including Body-Mass Index (BMI), waist circumference (lower than 88 cm, greater than or equal to 88 cm), blood pressure, blood glucose metabolism, and HDL-C. 

In particular, BMI values indicate 4 categories: (i) underweight, BMI < 18.50 kg/m^2^; (ii) normal weight, BMI 18.50 to 24.99 kg/m^2^; (iii) overweight, BMI 25.00 to 29.99 kg/m^2^; (iv) obesity, BMI ≥ 30.00 kg/m^2^. Regarding blood glucose levels, a physiological condition includes the following values: 80 to 100 mg/dL fasting, 70 to 200 mg/dL after eating, 120 to 140 mg/dL 2–3 h after eating. Impaired glucose tolerance (IGT) was defined as 101 to 125 mg/dL fasting, 190 to 230 mg/dL after eating, 140 to 160 mg/dL 2–3 h after eating; diabetic status was defined as 126 mg/dL fasting, 220 to 300 mg/dL after eating, and more than 200 mg/dL 2–3 h after eating. Concerning blood levels of HDL-C, values indicating low, normal, and high risk in women are higher than 60 mg/dL, between 50 and 59 mg/dL and less than 50 mg/dL, respectively. Blood pressure categories follow the American Heart Association Guidelines [37]. 

### 2.3. Statistical Analysis 

Heterogeneity among variables was tested using the Chi-squared test [38]. The Chi-squared test was used to compare continuous variables. Data were considered statistically significant with a *p*-value ≤ 0.05.

## 3. Results

We collected retrospective records of the medical questionnaire completed by the patients at the baseline (T0), before starting the assumption of the food supplement, and after 6 months (T1) of the dietary supplementation (Table 1). 

Regarding VMS, the oral administration of combined myo-Ins, cocoa polyphenols, and soy isoflavones significantly improved the investigated parameters. Specifically, after 6 months, the percentage of women perceiving 2–3 hot flushes significantly increased compared to the baseline (*p* value < 0.001) from 28% (T0) to 65% (T1), as a result of the percentage of women experiencing 4–9 hot flushes being strongly reduced from 54% to 18% (*p* value < 0.001). Concerning the severity of the hot flushes, at T0, 31% of patients had hot flushes of mild intensity, and 59% of them had moderate intensity. At T1, 62% of patients experienced mild intensity hot flushes (*p* value < 0.001), 27% experienced moderate intensity (*p* value < 0.001), with no more patients experiencing severe intensity of hot flushes (*p* value = 0.04). We obtained similar results in the case of reported sweats: data collected from women taking myo-Ins, cocoa polyphenols, and soy isoflavones revealed an improvement in terms of both frequency and extent of episodes. After 6 months of dietary supplementation, the percentage of women experiencing 2–3 episodes significantly increased (13% at T0 vs. 33% at T1, *p* value < 0.001), with no patients reporting more than 10 episodes (5% at T0 vs. 0% at T1, *p* value = 0.001). Furthermore, the severity of night sweats reduced: 87% of women perceived a moderate extent at T0, whereas 30% of them had a moderate extent at T1 (*p* value < 0.001). Simultaneously, 4% of women perceived a severe extent at T0, whereas none of them reported a severe entity at T1 (*p* value < 0.04).

Moreover, the administration of the aforementioned combined natural molecules demonstrated a positive correlation with a reduction in depression symptoms. At baseline, 87% of patients reported suffering from symptoms of depression of moderate extent at T0, which dropped to 56% at T1 (*p* value < 0.001). Concurrently, 4% of women revealed severe depression symptoms before starting the dietary supplementation, whereas none of them reported severe depression symptoms after 6 months (*p* value = 0.005). Consequently, the percentage of women experiencing a low extent of episodes of depression, or zero episodes of depression, significantly increased (*p* value < 0.001 for both).

Regarding metabolic profile, collected data revealed significant improvements after 6 months of oral administration of myo-Ins, cocoa polyphenols, and soy isoflavones. The number of women suffering from non-insulin-dependent diabetes (NIDD) reduced, by decreasing from 26% at T0 to 16% at T1 (*p* value = 0.04). High risk values for serum HDL-C were detected in 28% of women at T0 dropping to 17% of women at T1 (*p* value = 0.03). 

Concerning BMI, at T0 in our cohort of patients, only 7% of women exhibited a BMI in the normal range, while at T1 33% of women were of normal weight, with a significant reduction in the overweight and obesity women (from 88% to 51%, *p* value = 0.01 and from 14% to 9%, *p* value = 0.04). Following the oral administration, the number of women with waist circumferences lower than 88 cm increased, yet without reaching a statistical power. 

Finally, at baseline, 9% of patients exhibited elevated values of blood pressure, while at T1 only 3.5% suffered from elevated blood pressure, with a significant *p* value of 0.03.

## 4. Discussion

In this retrospective observational study, the oral administration of combined natural molecules including myo-Ins, cocoa polyphenols, and soy isoflavones was effective in improving both entity and frequency of VMS, including night sweats and hot flushes, and metabolic profile in menopausal women with MetS and climacteric disturbances.

Menopause is a physiological condition in women’s life, usually defined after a period of 12 consecutive months without a menstrual cycle. However, the hormonal changes that physiologically occur during menopause may expose women to several symptoms of various severity and frequency. Furthermore, during menopause, such hormonal changes may predispose women to an increased risk for developing metabolic disturbances such as obesity, insulin resistance, hyperinsulinemia with consequent dyslipidemia, and hypertension. These conditions are features of MetS, which may also increase the risk of cardiovascular disease [39]. In addition, the typical menopausal hormonal changes, such as the deficiency of estrogens, may contribute to the occurrence of other climacteric symptoms, including hot flushes and night sweats [40].

Hormonal therapy has been traditionally considered as the first line of treatment for the management of this condition. However, despite its efficacy in improving some menopausal symptoms, several concerns still remain regarding potential undesirable effects, which may reduce patient compliance [17]. For this reason, alternative approaches based on the use of natural molecules, such as the ones presented in the current study, succeed in obtaining a higher compliance due to their efficacy and improved safety profile. 

Soy extract has been demonstrated to counteract climacteric disorders and promote a balanced lipid metabolism. Specifically, a previous study demonstrated that soy proteins containing isoflavones significantly improve cardiovascular beneficial markers and adjusted cardiovascular risk after 6 months of supplementation in the early phase of menopause, in comparison to soy proteins without isoflavones, as evidenced by a significant decrease in metabolic features and systolic blood pressure [41]. Indeed, with the aim of implementing preventative measures against such conditions, acting also in the perimenopause period could provide a window of opportunity to improve women’s health and reduce the risk of developing cardiovascular diseases or obesity. With this perspective, alternative approaches to hormonal therapies, as dietary supplements or natural products, may become crucial strategies for also preventing following menopausal alterations by intervening in perimenopausal period. 

*Theobroma cacao* exerts a tonic vascular and metabolic effect, performs antioxidant functions, and regulates mood. Previous studies revealed beneficial effects on different clinical conditions including rheumatologic affections, such as fibromyalgia, in addition to degenerative diseases characterized by an oxidative stress injury [42,43,44]. A recent work reported that adding 10 g daily of cocoa-rich chocolate to the usual diet in menopausal patients decreases their percentage of body fat mass without changes to their overall bodyweight [45]. Moreover, cocoa polyphenols also demonstrated a mild improvement in cognitive ability and processing speed in the same patient population [46]. Considering the higher risk in menopause of developing cardiovascular diseases [47], a recent work by Grassi and colleagues demonstrated that cocoa polyphenols may contribute to an increase in the endothelial function, leading to improved arterial blood pressure, levels of cholesterol, and blood glucose, especially in elder women [31,32]. 

Considering the observed positive results of our supplementation on symptoms of depression and considering the association between healthy gut microbiota and mental health, including depression [48,49], it is noteworthy that cocoa polyphenols may exert prebiotic effects on microbiota [50]. Indeed, once reaching the intestine, cocoa polyphenols interact bidirectionally with the gut microbiota, and they are able to modulate its composition exerting prebiotic mechanisms [50], thus favoring the growth of beneficial microorganisms. In addition, previous preclinical studies suggested that soy isoflavones and genistein may also exert positive effects on depression-like behavior through the modulation of gut microbiota composition [51,52], alongside the beneficial effects on glucose and lipid dysmetabolism and inflammation.

Myo-Ins is a natural molecule widely used in restoring glucose and lipid metabolic disturbances. A previous study in post-menopausal women revealed that a 6-month oral supplementation of myo-Ins in combination with a balanced diet was able to improve blood pressure, HOMA index, and serum levels of cholesterol and triglycerides compared to the diet-alone control group [34]. Subsequently this study continued for a total of 12 months, where the evaluated parameters, aside from BMI and waist circumference, exhibited a significant improvement in the myo-Ins group compared to the control [35], thus opening a new promising approach in the management of menopause. 

Overall, the combination of myo-Ins, cocoa polyphenols, and soy isoflavones improved metabolic profile and related symptoms in menopausal women. A previous study by D’Anna and colleagues also revealed a protective action of such combined natural molecules on cardiac markers in menopausal patients affected by MetS, following the administration of the same supplement of this study [36]. 

For the first time, this retrospective study describes the positive effects of the combined action of myo-Ins, cocoa polyphenols, and soy isoflavones on VMS and the depression-like symptoms that are characteristic of menopausal women, thus investigating their potential clinical value. Taken together, these natural molecules succeed in improving the menopausal state, reducing vasomotor manifestations in terms of severity and frequency of episodes of hot flushes, and improving the metabolic profile in the study population. In particular, the improvement of BMI, waist circumference, blood pressure, and serum levels of glucose and HDL correlate with a reduced risk of developing cardiovascular complications. Furthermore, they positively impact depression-like symptoms which are frequent in menopause, affecting up to 70% of women, by also supporting a potential microbiota-mediated effect.

It is crucial to bear in mind that the successful management of menopause depends on the prescription of tailored therapies to the individual patient. For example, women who poorly respond to HRT or who have concerns about their use may be the best candidates for alternative natural molecule-based approaches as natural hormone replacement therapies [38]. Limitations of this clinical study include its retrospective nature and the lack of a control group; furthermore, data were analyzed upon self-reported data by patients. In the future, larger prospective clinical studies with a control group are necessary to corroborate the efficacy of such natural molecules as a step forward in the management of menopause. 

## 5. Conclusions

The oral administration of combined myo-Ins, cocoa polyphenols, and soy isoflavones has been shown to be a valid strategy as natural replacement therapy for improving menopausal symptoms, including VMS, symptoms of depression, and metabolic dysfunctions, thus also reducing cardiovascular risk in menopausal women with MetS. Of course, further prospective clinical trials on a wider population with a control group are required to corroborate the observed results on menopausal symptoms.

## Figures and Tables

**Table 1:** Distribution of menopausal manifestations at the beginning of dietary supplement administration (T0) and after 6 months (T1).

Parameters	T0	T1	*p* Value
Hot flushes, No. (%)			
None	12 (6)	22 (11)	0.07
2–3	56 (28)	130 (65)	<0.001 *
4–9	108 (54)	36 (18)	<0.001 *
>10	24 (12)	12 (6)	0.055
Severity of hot flushes, No. (%)			
None	16 (8)	24 (12)	0.22
Mild	62 (31)	124 (62)	<0.001 *
Moderate	118 (59)	54 (27)	<0.001 *
Severe	4 (2)	0 (0)	0.04 *
Sweats, No. (%)			
None	8 (4)	18 (9)	0.057
2–3	26 (13)	66 (33)	<0.001 *
4–9	156 (78)	116 (58)	0.06
>10	10 (5)	0 (0)	<0.001 *
Severity of sweats, No. (%)			
None	0 (0)	28 (14)	<0.001 *
Mild	10 (5)	112 (56)	<0.001 *
Moderate	186 (87)	60 (30)	<0.001 *
Severe	4 (4)	0 (0)	0.04 *
Depressive symptoms			
None	0 (0)	24 (12)	<0.001 *
Mild	18 (9)	64 (32)	<0.001 *
Moderate	174 (87)	112 (56)	<0.001 *
Severe	8 (4)	0 (0)	0.005 *
BMI, No. (%)			
Underweight	6 (3)	14 (7)	0.08
Normal weight	14 (7)	66 (33)	<0.001 *
Overweight	154 (88)	102 (51)	0.01 *
Obesity	28 (14)	18 (9)	0.04 *
Waist circumference			
Lower than 88 cm	78 (39)	96 (48)	0.25
Greater than or equal to 88 cm	122 (61)	104 (52)	0.33
Blood pressure, No. (%)			
Low	6 (3)	3 (1.5)	0.32
Normal	175 (87.5)	190 (95)	0.57
Elevated	18 (9)	7 (3.5)	0.03 *
High	1 (0.5)	0 (0)	0.31
Blood glucose			
Normal	98 (48)	133 (66.5)	0.051
IGT	46 (23)	31 (15.5)	0.11
IDD	6 (3)	4 (2)	0.53
NIDD	52 (26)	32 (16)	0.04 *
HDL Cholesterol, No. (%)			
Low risk	18 (9)	10 (5)	0.14
Normal	126 (63)	156 (78)	0.17
High risk	56 (28)	34 (17)	0.03 *

Collected retrospective data through the medical questionnaires completed by patients at baseline (T0) and after 6 months (T1) of the dietary assumption. BMI: body-mass index; IGT: impaired glucose tolerance; IDD: insulin-dependent diabetes; NIDD: non-insulin-dependent diabetes. * Statistically significant analysis (*p* value < 0.05).

## Data Availability

The datasets generated and analyzed during this study are available from the corresponding author upon reasonable request.

## References

[1] Johnson A., Roberts L., Elkins G. (2019). Complementary and Alternative Medicine for Menopause. J. Evid. Based Integr. Med..

[2] Sussman M., Trocio J., Best C., Mirkin S., Bushmakin A.G., Yood R., Friedman M., Menzin J., Louie M. (2015). Prevalence of menopausal symptoms among mid-life women: Findings from electronic medical records. BMC Womens Health.

[3] Sarri G., Davies M., Lumsden M.A., Guideline Development G. (2015). Diagnosis and management of menopause: Summary of NICE guidance. BMJ.

[4] Takahashi T.A., Johnson K.M. (2015). Menopause. Med. Clin. N. Am..

[5] Lobo R.A., Davis S.R., De Villiers T.J., Gompel A., Henderson V.W., Hodis H.N., Lumsden M.A., Mack W.J., Shapiro S., Baber R.J. (2014). Prevention of diseases after menopause. Climacteric.

[6] Sobenin I.A., Myasoedova V.A., Orekhov A.N. (2016). Phytoestrogen-Rich Dietary Supplements in Anti-Atherosclerotic Therapy in Postmenopausal Women. Curr. Pharm. Des..

[7] Thaung Zaw J.J., Howe P.R.C., Wong R.H.X. (2018). Postmenopausal health interventions: Time to move on from the Women’s Health Initiative?. Ageing Res. Rev..

[8] El Khoudary S.R., Ceponiene I., Samargandy S., Stein J.H., Li D., Tattersall M.C., Budoff M.J. (2018). HDL (High-Density Lipoprotein) Metrics and Atherosclerotic Risk in Women. Arterioscler. Thromb. Vasc. Biol..

[9] Thurston R.C., Sutton-Tyrrell K., Everson-Rose S.A., Hess R., Matthews K.A. (2008). Hot flashes and subclinical cardiovascular disease: Findings from the Study of Women’s Health Across the Nation Heart Study. Circulation.

[10] Avis N.E., Crawford S.L., Greendale G., Bromberger J.T., Everson-Rose S.A., Gold E.B., Hess R., Joffe H., Kravitz H.M., Tepper P.G. (2015). Duration of menopausal vasomotor symptoms over the menopause transition. JAMA Intern. Med..

[11] Dennerstein L., Lehert P., Burger H.G., Guthrie J.R. (2007). New findings from non-linear longitudinal modelling of menopausal hormone changes. Hum. Reprod. Update.

[12] Grady D. (2006). Clinical practice. Management of menopausal symptoms. N. Engl. J. Med..

[13] Santoro N., Epperson C.N., Mathews S.B. (2015). Menopausal Symptoms and Their Management. Endocrinol. Metab. Clin. N. Am..

[14] Utian W.H. (2005). Psychosocial and socioeconomic burden of vasomotor symptoms in menopause: A comprehensive review. Health Qual. Life Outcomes.

[15] Peacock K., Ketvertis K.M. (2018). Menopause.

[16] Zhu D., Chung H.F., Dobson A.J., Pandeya N., Anderson D.J., Kuh D., Hardy R., Brunner E.J., Avis N.E., Gold E.B. (2020). Vasomotor menopausal symptoms and risk of cardiovascular disease: A pooled analysis of six prospective studies. Am. J. Obstet. Gynecol..

[17] Mehta J., Kling J.M., Manson J.E. (2021). Risks, Benefits, and Treatment Modalities of Menopausal Hormone Therapy: Current Concepts. Front. Endocrinol..

[18] Maclennan A.H., Broadbent J.L., Lester S., Moore V. (2004). Oral oestrogen and combined oestrogen/progestogen therapy versus placebo for hot flushes. Cochrane Database Syst. Rev..

[19] Posadzki P., Lee M.S., Moon T.W., Choi T.Y., Park T.Y., Ernst E. (2013). Prevalence of complementary and alternative medicine (CAM) use by menopausal women: A systematic review of surveys. Maturitas.

[20] Rossouw J.E., Anderson G.L., Prentice R.L., LaCroix A.Z., Kooperberg C., Stefanick M.L., Jackson R.D., Beresford S.A., Howard B.V., Johnson K.C. (2002). Risks and benefits of estrogen plus progestin in healthy postmenopausal women: Principal results From the Women’s Health Initiative randomized controlled trial. JAMA.

[21] Ma J., Drieling R., Stafford R.S. (2006). US women desire greater professional guidance on hormone and alternative therapies for menopause symptom management. Menopause.

[22] Liu Z.M., Ho S.C., Chen Y.M., Woo J. (2013). A six-month randomized controlled trial of whole soy and isoflavones daidzein on body composition in equol-producing postmenopausal women with prehypertension. J. Obes..

[23] Chen L.R., Chen K.H. (2021). Utilization of Isoflavones in Soybeans for Women with Menopausal Syndrome: An Overview. Int. J. Mol. Sci..

[24] Su B.Y., Tung T.H., Chien W.H. (2018). Effects of Phytoestrogens on Depressive Symptoms in Climacteric Women: A Meta-Analysis of Randomized Controlled Trials. J. Altern. Complement. Med..

[25] Pintova S., Dharmupari S., Moshier E., Zubizarreta N., Ang C., Holcombe R.F. (2019). Genistein combined with FOLFOX or FOLFOX-Bevacizumab for the treatment of metastatic colorectal cancer: Phase I/II pilot study. Cancer Chemother. Pharmacol..

[26] Thangavel P., Puga-Olguin A., Rodriguez-Landa J.F., Zepeda R.C. (2019). Genistein as Potential Therapeutic Candidate for Menopausal Symptoms and Other Related Diseases. Molecules.

[27] Yu L., Rios E., Castro L., Liu J., Yan Y., Dixon D. (2021). Genistein: Dual Role in Women’s Health. Nutrients.

[28] Kwon S.H., Kang M.J., Huh J.S., Ha K.W., Lee J.R., Lee S.K., Lee B.S., Han I.H., Lee M.S., Lee M.W. (2007). Comparison of oral bioavailability of genistein and genistin in rats. Int. J. Pharm..

[29] Jaimez R.E., Barragan L., Fernandez-Nino M., Wessjohann L.A., Cedeno-Garcia G., Sotomayor Cantos I., Arteaga F. (2022). Theobroma cacao L. cultivar CCN 51: A comprehensive review on origin, genetics, sensory properties, production dynamics, and physiological aspects. PeerJ.

[30] Grassi D., Lippi C., Necozione S., Desideri G., Ferri C. (2005). Short-term administration of dark chocolate is followed by a significant increase in insulin sensitivity and a decrease in blood pressure in healthy persons. Am. J. Clin. Nutr..

[31] Grassi D., Necozione S., Lippi C., Croce G., Valeri L., Pasqualetti P., Desideri G., Blumberg J.B., Ferri C. (2005). Cocoa reduces blood pressure and insulin resistance and improves endothelium-dependent vasodilation in hypertensives. Hypertension.

[32] Tangney C.C., Rasmussen H.E. (2013). Polyphenols, inflammation, and cardiovascular disease. Curr. Atheroscler. Rep..

[33] Bevilacqua A., Bizzarri M. (2018). Inositols in Insulin Signaling and Glucose Metabolism. Int. J. Endocrinol..

[34] Giordano D., Corrado F., Santamaria A., Quattrone S., Pintaudi B., Di Benedetto A., D’Anna R. (2011). Effects of myo-inositol supplementation in postmenopausal women with metabolic syndrome: A perspective, randomized, placebo-controlled study. Menopause.

[35] Santamaria A., Giordano D., Corrado F., Pintaudi B., Interdonato M.L., Vieste G.D., Benedetto A.D., D’Anna R. (2012). One-year effects of myo-inositol supplementation in postmenopausal women with metabolic syndrome. Climacteric.

[36] D’Anna R., Santamaria A., Cannata M.L., Interdonato M.L., Giorgianni G.M., Granese R., Corrado F., Bitto A. (2014). Effects of a new flavonoid and Myo-inositol supplement on some biomarkers of cardiovascular risk in postmenopausal women: A randomized trial. Int. J. Endocrinol..

[37] Whelton P.K., Carey R.M., Aronow W.S., Casey D.E., Collins K.J., Dennison Himmelfarb C., DePalma S.M., Gidding S., Jamerson K.A., Jones D.W. (2018). 2017 ACC/AHA/AAPA/ABC/ACPM/AGS/APhA/ASH/ASPC/NMA/PCNA Guideline for the Prevention, Detection, Evaluation, and Management of High Blood Pressure in Adults: A Report of the American College of Cardiology/American Heart Association Task Force on Clinical Practice Guidelines. J. Am. Coll. Cardiol..

[38] Chaimani A., Higgins J.P., Mavridis D., Spyridonos P., Salanti G. (2013). Graphical tools for network meta-analysis in STATA. PLoS ONE.

[39] Isomaa B., Almgren P., Tuomi T., Forsen B., Lahti K., Nissen M., Taskinen M.R., Groop L. (2001). Cardiovascular morbidity and mortality associated with the metabolic syndrome. Diabetes Care.

[40] Taechakraichana N., Jaisamrarn U., Panyakhamlerd K., Chaikittisilpa S., Limpaphayom K.K. (2002). Climacteric: Concept, consequence and care. J. Med. Assoc. Thai..

[41] Sathyapalan T., Aye M., Rigby A.S., Thatcher N.J., Dargham S.R., Kilpatrick E.S., Atkin S.L. (2018). Soy isoflavones improve cardiovascular disease risk markers in women during the early menopause. Nutr. Metab. Cardiovasc. Dis..

[42] Islam M.A., Alam F., Solayman M., Khalil M.I., Kamal M.A., Gan S.H. (2016). Dietary Phytochemicals: Natural Swords Combating Inflammation and Oxidation-Mediated Degenerative Diseases. Oxid. Med. Cell Longev..

[43] Okamoto T., Kobayashi R., Natsume M., Nakazato K. (2016). Habitual cocoa intake reduces arterial stiffness in postmenopausal women regardless of intake frequency: A randomized parallel-group study. Clin. Interv. Aging.

[44] Garcia-Yu I.A., Garcia-Ortiz L., Gomez-Marcos M.A., Alonso-Dominguez R., Gonzalez-Sanchez J., Mora-Simon S., Gonzalez-Manzano S., Rodriguez-Sanchez E., Maderuelo-Fernandez J.A., Recio-Rodriguez J.I. (2018). Vascular and cognitive effects of cocoa-rich chocolate in postmenopausal women: A study protocol for a randomised clinical trial. BMJ Open.

[45] Garcia-Yu I.A., Garcia-Ortiz L., Gomez-Marcos M.A., Rodriguez-Sanchez E., Lugones-Sanchez C., Maderuelo-Fernandez J.A., Recio-Rodriguez J.I. (2021). Cocoa-rich chocolate and body composition in postmenopausal women: A randomised clinical trial. Br. J. Nutr..

[46] Garcia-Yu I.A., Garcia-Ortiz L., Gomez-Marcos M.A., Rodriguez-Sanchez E., Mora-Simon S., Maderuelo-Fernandez J.A., Recio-Rodriguez J.I. (2022). Effects of cocoa-rich chocolate on cognitive performance in postmenopausal women. A randomised clinical trial. Nutr. Neurosci..

[47] Agrinier N., Cournot M., Dallongeville J., Arveiler D., Ducimetiere P., Ruidavets J.B., Ferrieres J. (2010). Menopause and modifiable coronary heart disease risk factors: A population based study. Maturitas.

[48] Limbana T., Khan F., Eskander N. (2020). Gut Microbiome and Depression: How Microbes Affect the Way We Think. Cureus.

[49] Irum N., Afzal T., Faraz M.H., Aslam Z., Rasheed F. (2023). The role of gut microbiota in depression: An analysis of the gut-brain axis. Front. Behav. Neurosci..

[50] Sorrenti V., Ali S., Mancin L., Davinelli S., Paoli A., Scapagnini G. (2020). Cocoa Polyphenols and Gut Microbiota Interplay: Bioavailability, Prebiotic Effect, and Impact on Human Health. Nutrients.

[51] Yang R., Jia Q., Mehmood S., Ma S., Liu X. (2021). Genistein ameliorates inflammation and insulin resistance through mediation of gut microbiota composition in type 2 diabetic mice. Eur. J. Nutr..

[52] Wang L., Wu X., Ma Y., Li X., Zhang J., Zhao L. (2021). Supplementation with soy isoflavones alleviates depression-like behaviour via reshaping the gut microbiota structure. Food Funct..

